# Role of the Gene *tri14* in Biosynthesis of the Trichothecene Toxin Harzianum A in *Trichoderma arundinaceum*

**DOI:** 10.3390/toxins17090427

**Published:** 2025-08-26

**Authors:** Natalia Martínez-Reyes, Rosa E. Cardoza, Susan P. McCormick, Guixia Hao, Joaquín Rodríguez-Fernández, Robert H. Proctor, Santiago Gutiérrez

**Affiliations:** 1 Grupo Universitario de Investigación en Ingeniería y Agricultura Sostenible (GUIIAS), Área de Microbiología, Universidad de León, 24400 Ponferrada, Spain; nmarr@unileon.es (N.M.-R.); re.cardoza@unileon.es (R.E.C.); jrodf@unileon.es (J.R.-F.); 2 United States Department of Agriculture, Agricultural Research Service, National Center for Agricultural Utilization Research, Mycotoxin Prevention and Applied Microbiology, 1815 N University St., Peoria, IL 61604, USA; susan.mccormick@usda.gov (S.P.M.); guixia.hao@usda.gov (G.H.); robert.proctor@usda.gov (R.H.P.)

**Keywords:** trichothecene biosynthesis, biological activity, *tri14*, antifungal activity, self-resistance, resistance to ROS

## Abstract

Trichothecenes are a family of toxic metabolites produced by multiple fungal species. All trichothecene analogs include an epoxide-containing tricyclic structure known as 12,13-epoxytrichothec-9-ene (EPT) but differ by the presence, absence and types of substituents attached to EPT. Among the 21 known genes associated with trichothecene biosynthesis, *tri14* is one of only three that are universally found in all trichothecene-producing fungi. Recent studies have revealed that the *tri14*-encoded protein, Tri14, enhances the biosynthetic reaction that forms EPT, a reaction previously thought to occur spontaneously. In our study, we assessed the impact of *tri14* deletion on the biology of *Trichoderma arundinaceum*, a producer of the trichothecene harzianum A (HA). The results revealed that *tri14* deletion reduced HA production by 69%, an outcome that was associated with diminished antifungal activity. To our knowledge, this is the first study showing that *tri14* is required for wild-type production of a trichothecene analog by a fungal organism. *tri14* deletion also had moderate effects on the expression of some other trichothecene biosynthetic genes, as well as in the production of metabolites beyond HA. These results suggest that Tri14 plays a crucial role in EPT formation, leading to diverse downstream effects on the biology of *T. arundinaceum*.

## 1. Introduction

Trichothecenes are a family of sesquiterpenoid toxins produced by fungi from at least three fungal classes, including species of *Aspergillus*, *Fusarium*, *Isaria*, *Microcyclospora*, *Paramyrothecium*, *Peltaster*, *Stachybotrys*, *Trichothecium* and *Trichoderma*. Collectively, these fungi produce over 150 structurally distinct analogs of trichothecenes, but individual species tend to produce only a few analogs, and one analog is often produced at markedly higher levels than the others [1,2]. All trichothecene analogs have a tricyclic skeleton known as 12,13-epoxytrichothec-9-ene (EPT). The analogs differ from one another by the presence, absence and types of substituents attached to EPT. Much of what is known about the biochemistry and genetics of trichothecene biosynthesis has been derived from studies of four trichothecene-producing species: *Fusarium graminearum*, *F. sporotrichioides*, *Paramyrothecium roridum* and *Trichoderma arundinaceum* [2,3,4,5,6]. These studies have identified 21 trichothecene biosynthetic genes (*tri*), and in most cases, the roles of the genes in trichothecene biosynthesis have been described. *tri* genes typically occur adjacent to one another in a trichothecene biosynthetic gene cluster, but in some species, the genes are distributed over two clusters and/or a single *tri* gene can be located in a genomic region that lacks other known *tri* genes [2,4].

*Trichoderma* species occur in diverse habitats, and some species are used as or have potential as biological control agents. Studies on the metabolism, genetics and ecology of these fungi are driven in part by the idea that the results will provide insights into how to improve the biological control conferred by *Trichoderma*. Over the past two decades, numerous studies have demonstrated that multiple *Trichoderma* species can produce trichothecenes [7,8,9,10,11,12]. Species from at least three *Trichoderma* lineages (the *Brevicompactum*, *Psychrophila* and *Rubi* clades) can produce a subclass of trichothecene analogs known as simple trichothecenes because they lack a macrocyclic ring structure [4]. These analogs include harzianum A (HA), trichodermol and trichodermin. Species in at least one *Trichoderma* lineage (*Psychrophila* clade) can produce trichothecenes with a macrocyclic ring (i.e., macrocyclic trichothecenes) such as roridin A and E. In the *Trichoderma* species that have been examined, *tri* genes are distributed over three genomic regions: one region has *tri5* but no other known *tri* genes; a second region has *tri14*, *tri12*, *tri22*, *tri10*, *tri3*, *tri4* and *tri6*, which are arranged contiguously in the order indicated; and a third region has *tri18*, *tri23* and *tri17*, which are also arranged contiguously [2,4]. There are exceptions to this arrangement. For example, *T. brevicompactum* lacks functional homologs of *tri17* and *tri23*, which are required for the formation of a linear 8-carbon substituent sterified at carbon atom 4 (C4), which is found in HA. As a result, *T. brevicompactum* does not produce HA and instead produces trichodermin (4-acetyl EPT) [13,14]. Production of HA by *T. arundinaceum* has served as a model system for understanding the biochemistry and genetics of trichothecene biosynthesis in *Trichoderma* [4]. In addition, the production of HA and polyketide-derived aspinolides can impact biological control activity of *T. arundinaceum* [4].

To our knowledge, *tri3*, *tri5* and *tri14* are the only *tri* genes that occur in all trichothecene-producing species [2,15]. Gene function analyses indicate that during trichothecene biosynthesis, the *tri5*-encoded terpene synthase (Tri5) catalyzes cyclization of farnesyl diphosphate to trichodiene, the terpene parent compound of all trichothecene analogs [2,16]. Despite multiple studies, the role of *tri14* in trichothecene biosynthesis was not resolved until recently in a study that included both cell-free, and heterologous expression analyses of *tri3* and *tri14* [17]. These studies indicated that the *tri3*-encoded acetyltransferase (Tri3) catalyzes 11-*O*-acetylation of early trichothecene intermediates, isotrichodiol in some fungi and isotrichotriol in others, and that Tri14 is an enzyme with a previously undescribed activity that catalyzes the cyclization of the resulting acetylated intermediates to form 12,13-epoxythichothec-9-ene (EPT) and 3-hydroxy EPT (isotrichodermol), respectively. Prior to these analyses, cyclization of isotrichodiol and isotrichodiol to EPT and 3-hydroxy EPT, respectively, was thought to occur spontaneously [2,4]. Despite these recent discoveries, prior studies also indicated that Tri3 catalyzes the 4- or 11-*O*-acetylation of EPT-derived trichothecene biosynthetic intermediates [14,18,19,20,21]. In addition, previous studies have indicated that *tri14* gene is not required for the formation of trichothecenes in *F. graminearum* and *Trichoderma brevicompactum* [15,22,23]. However, some of these studies have also revealed that *tri14* contributes to the pathogenesis of *F. graminearum* in wheat. The latter finding is notable because the ability of *F. graminearum* to produce trichothecenes contributes to its aggressiveness in wheat [24,25,26]. In addition, *tri14* deletion affected the expression of some other *tri* genes in *T. brevicompactum* [23] and the oxidative stress response in *F. graminearum* [15].

In light of the newly discovered role of *tri14* in trichothecene biosynthesis, the objective of the current study was to determine how *tri14* impacts *T. arundinaceum* trichothecene production as well as other selected downstream biological processes, namely biological control activity, *tri* gene expression, self-protection from the toxic effects of HA and farnesol, and response to oxidative stress in *T. arundinaceum*. The results of the analyses indicate *tri14* is required for wild-type levels of HA production and the biological control activity of *T. arundinaceum*. To our knowledge, this is the first study showing that deletion of *tri14* changes the trichothecene production phenotype of a fungus in culture.

## 2. Results

### 2.1. T. arundinaceum tri14 Deletion

**Figure 1:**
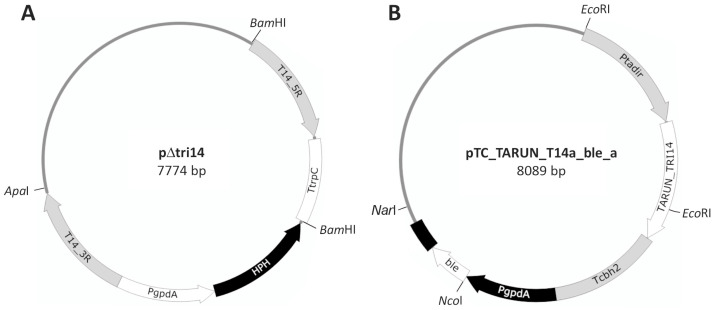
(**A**) Scheme of pΔtri14 plasmid. T14_5R and T14_3R are the regions located at the 5′ and 3′ sides of *T. arundinaceum tri14* gene. PgpdA—promoter region of the *Aspergillus nidulans* glyceraldehyde-3-phosphate dehydrogenase; TtrpC—*A. nidulans* trpC terminator region; HPH—*E. coli* hygromycin B resistance gene. (**B**) Map of plasmid pTCS_TARUN_T14a_ble_a designed to express *T. arundinaceum tri14* gene. *ble*—phleomycin/bleomycin resistance gene from *Streptoalloteichus hindustanus*. Ptadir indicates the promoter region of the *Trichoderma harzianum tadir* gene, and Tcbh2 indicates the transcriptional terminator of the *T. reesei* cellobiohydrolase 2 encoding gene.

**Figure 2:**
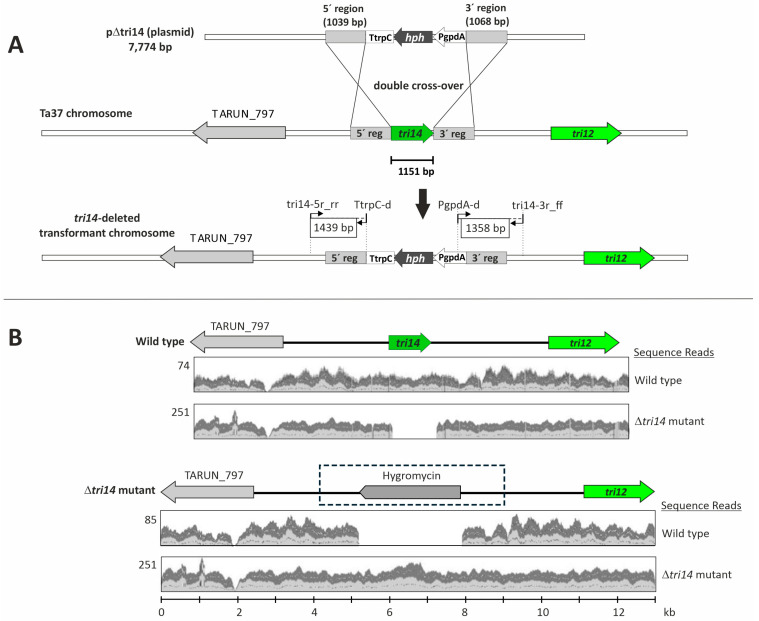
Strategy for *T. arundinaceum tri14* deletion. (**A**) Expected construct obtained as result of the double recombination between the *tri14* flanking extremes in pΔtri14 and in the *T. arundinaceum* genome. Oligonucleotides tri14-5r_rr and tri14-3_ff are described in Table S1, and oligonucleotides TrpC-d and PgpdA-d sequences have been previously described [18]. (**B**) Confirmation of *tri14* deletion in *T. arundinaceum* strain Δtri14.10 by in silico mapping of whole-genome sequence reads to reference sequences. The reference sequences (**wild-type** and **Δ*tri14* mutant**) are shown at the top of the upper and lower panels, and mapped reads are depicted as jagged and undulating gray areas within the two rectangles below each reference sequence. For the mapped reads, the two shades of gray indicate reads in forward and reverse orientations. Reference sequences: **Wild-type**—11.4 kb segment in *tri14* region of the wild-type progenitor strain (Ta37) of *T. arundinaceum*. **Δ*tri14* mutant**—13 kb segment predicted to result from deletion of the *tri14* coding region by transformation of Ta37 with deletion plasmid pΔtri14. In the reference sequences, genes are indicated by arrows that point in the direction of transcription. The label Hygromycin indicates the hygromycin resistance gene (HPH); and the label TAURN_797 indicates the gene predicted to encode α-N-acetylglucosaminidase (GenBank accession RFU81391.1). The rectangle outlined with broken lines and surrounding part of reference sequence of **Δ*tri14* mutant** indicates the segment of DNA homologous to the deletion cassette in plasmid pΔtri14. Genome sequence reads used for mapping are indicated to the right of each panel. The sequence reads were generated from the wild-type progenitor (wild-type) strain Ta37 and the Δ*tri14* mutant strain Δtri14.10. Numbers to the left of rectangles with mapped reads indicate maximum read coverage. The marked differences in maximum coverage values for the wild-type and Δ*tri14* mutant likely resulted from the different methods used to generate sequence data for the two strains. The mapping analysis was performed using the Map Reads to Reference function in CLC Genomics Workbench 24. Note that green colors are used to point *tri14* and *tri12* genes.

Transformation of *T. arundinaceum* protoplasts with plasmid pΔtri14 (Figure 1) resulted in the isolation of 12 stable transformants that retained their hygromycin-resistant phenotype after the selection process. These transformants were analyzed by PCR with oligonucleotides tri14_5R_rr/TtrpC-d and tri14_3r_ff/PgpdA-d, which were designed to amplify the 1439 bp and 1358 bp fragments corresponding to the 5′ and 3′ regions, respectively. These amplicons were expected to occur when the *tri14* coding region was deleted as a result of double recombination between pΔtri14 and the *tri14*-flanking regions in *T. arundinaceum*. Only one transformant (mutant strain Δtri14.10) yielded the expected PCR amplicons (Figure S1), and it was selected for further studies. The nucleotide sequences of the two PCR amplicons from Δtri14.10 matched the sequences expected to result from the double recombination event (Figure 2). An analysis of the genome sequence of Δtri14.10 revealed that only one copy of the deletion cassette had integrated in the *tri14* region but not at other genomic locations. Thus, both the PCR and genome sequence analyses indicate that the *tri14* coding region was deleted in strain Δtri14.10 (Figure 2 and Figure S1).

### 2.2. Effect of tri14 Deletion on HA Production

**Figure 3:**
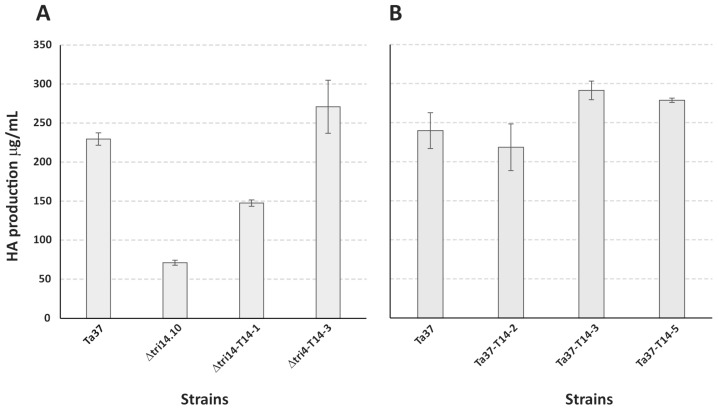
HA production in cultures of selected *Trichoderma arundinaceum* strains from two experiments (**A**,**B**). (**A**): Ta37: wild-type progenitor strain; Δtri14.10: *tri14* deletion mutant; Δtri14-T14-1 and Δtri14-T14-3: transformants of Δtri14.10 carrying the *tri14* expression cassette. (**B**): Ta37: wild-type progenitor strain; Ta37-T14-2, Ta37-T14-3 and Ta37-T14-5: transformants of Ta37 carrying the *tri14* expression cassette. Values are micrograms of harzianum A (HA) per mL of culture filtrate.

An analysis of 48 h PDB cultures of Δtri14.10 and the wild-type progenitor strain revealed that *tri14* deletion reduced the level of HA produced by 69% (Table 1, Figure 3A). This result contrasts findings that *tri14* deletion in *F. graminearum* did not significantly affect trichothecene production in cultures of the fungus [15,22].

### 2.3. Isolation of Δtri14 Add-Back and Ta37 Transformants Overexpressing T. arundinaceum tri14 Gene

The transformation of Δtri14.10 protoplasts with the linearized *tri14* overexpression plasmid pTC_TARUN_T14a_ble_a (Figure 1) yielded 25 transformants. After the phleomycin resistance selection process, six transformants that retained resistance were randomly selected for PCR analysis with primers T14_IF_Ct/T14_IF_Nt to confirm that the *tri14* overexpression cassette had integrated into the transformants’ genomes. In the PCR analysis, five of the transformants yielded the expected 820 bp fragment (Figure S2A; Table S1) and were selected for further analyses. The transformation of Ta37 protoplasts with the linearized *tri14* expression plasmid yielded more than 100 transformants. Ten of the transformants were analyzed by PCR as described above. Nine of the Ta37 transformants yielded the 993 bp PCR fragment expected using the oligonucleotides tri14_IF_Ct/Ptadir (Figure S2B, Table S1).

### 2.4. HA Production in Strains Overexpressing tri14 Gene

To assess the effect of the presence of the *tri14* expression cassette in Δtri14.10, the five selected add-back transformants were grown in PDB medium for 48 h at 28 °C and 250 rpm, and then the levels of HA in the resulting culture filtrates were analyzed by HPLC. Four of the five transformants produced significantly higher levels of HA (between 147.58 and 270.94 μg/mL) than Δtri14.10 (71.16 μg/mL) (Figure 3; Table 1 and Table S2). To assess the effect of the presence of the *tri14* overexpression cassette in Ta37 (wild-type strain), three Ta37 transformants carrying the overexpression plasmid were grown and analyzed for HA production as indicated above. The levels of HA (219–291 μg/mL) in the culture filtrates of the transformants spanned the levels in the culture filtrates of Ta37 (240 μg/mL) (Figure 3; Table 2). Thus, transformation of Ta37 with the *tri14* overexpression plasmid did not consistently increase the levels of HA production in the resulting transformants.

### 2.5. Effect of Deletion and Overexpression of tri14 on Expression of Other Tri Genes

**Figure 4:**
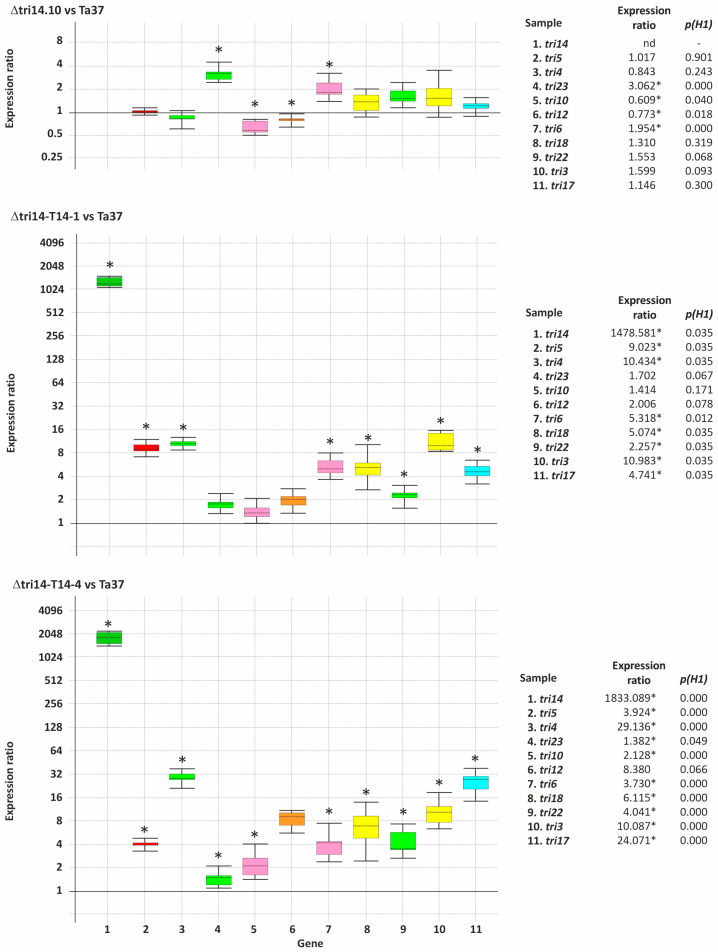
RT-qPCR analysis of *tri* gene expression levels in ∆tri14.10 (*tri14* mutant), ∆tri14-T14.1 and ∆tri14-T14-4 (add-back *tri14* mutants) compared to the reference strain Ta37 (wild type). Boxes are illustrated in different colors depending on the gene function. Red: terpene synthase (*tri5*); dark green: *tri14*; light green: monooxygenases (*tri4*, *tri23* and *tri22*); pink: transcription factors (*tri6* and *tri10*); brown: transporter (*tri12*); yellow: acyl/acetyltransferase (*tri18* and *tri3*); blue: polyketide synthase (*tri17*). Expression ratios were calculated as described previously [27,28]. Transcription levels are ratios calculated relative to the wild-type strain (Ta37). Statistically significant values (p(H1) < 0.05) are indicated with an asterisk at the right of each panel and in the upper part of each graph.

Expression of all *T. arundinaceum tri* genes was analyzed by quantitative PCR from mycelia grown in PDB medium for 48 h. Values were calculated as expression ratios in the strains assayed versus the levels observed in Ta37 (wild-type strain). Thus, *tri14* deletion resulted in only slight changes in the level of expression of the other *tri* genes. The highest differential expression observed was for *tri23*, with an expression ratio of 3.062 (*p* = 0.000) fold versus the level of expression in Ta37 (Figure 4).

In the *tri14* deletion mutant, the expression of *tri14* was undetectable. In the *tri14* add-back strains, Δtri14-T14-1 and Δtri14-T14-4, the expression of *tri14* was markedly increased relative to Ta37. The ratios of expression of *tri14* gene in the two *tri14* add-back transformants were 1478.6 (*p*= 0.03) and 1833.1 (*p* = 0.00) fold, respectively. Further, overexpression of the *tri14* gene in the add-back transformants also resulted in upregulation of most other *tri* genes. Thus, in transformant Δtri14-T14-1, the expression ratios ranged from 1.4 (*p* = 0.17) to 10.9 (*p* = 0.03) fold, for *tri10* and *tri3* genes, respectively, while in the transformant Δtri14-T14-4, the expression ratios ranged from 1.4 (*p* = 0.05) to 29.1 (*p* = 0.00) fold, for *tri23* and *tri4* genes, respectively (Figure 4).

### 2.6. Antifungal Activity

**Figure 5:**
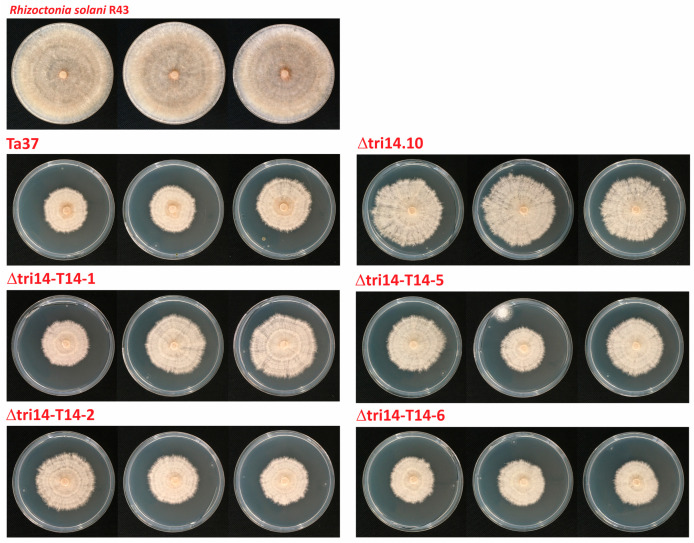
Antifungal activity against *R. solani* of the wild-type (Ta37), *tri14* deletion mutant (Δtri14.10) and *tri14* add-back (Δtri14-T14-1, Δtri14-T14-2, Δtri14-T14-5 and Δtri14-T14-6) strains. Plates were incubated for 15 days after removal the cellophane membrane and the *Trichoderma* plug, and the disposal of *R. solani* plug.

The wild-type strain Ta37, the *tri14* deletion mutant Δtri14.10 and five *tri14* add-back transformants were analyzed for their antifungal activity against the fungal phytopathogen *Rhizoctonia solani* R43 using a membrane assay. The results of the assay indicated that *tri14* deletion reduced the inhibitory activity of *T. arundinaceum*. That is, the antifungal activity of the *tri14* mutant was reduced by 43.67% relative to the wild type (Figure 5, Table S3). Collectively, the *tri14* add-back transformants exhibited a significant variation in antifungal activity against *R. solani*. In general, however, the add-back transformants exhibited antifungal activity that was greater than that of the deletion mutant and similar to that of the wild-type strain (Figure 5, Table S3). Overall, the levels of antifungal activity were positively correlated with the levels of HA produced by the *T. arundinaceum* strains.

### 2.7. In Silico Analysis of Tri14

To investigate potential functions of the Tri14 protein, structural analyses were performed on the predicted amino acid sequences of Tri14 orthologs from *T. arundinaceum*, *T. brevicompactum*, *Fusarium graminearum* and *F. sporotrichioides* using the program AlphaFold (https://alphafold.ebi.ac.uk accessed on 9 October 2022). All orthologs had an abundance of beta sheets in their predicted secondary structure and a barrel conformation in their tertiary structure (Figure S3). The abundance of β-lamina is typical for fatty-acid-binding proteins. Furthermore, a search for conserved regions in Tri14 led to the detection of a CAAX domain (one copy in *Fusarium* Tri14 homologs and two copies in *Trichoderma* Tri14 homologs) that is associated with the prenylation of proteins [29], including the binding of farnesyl diphosphate (farnesylation) and geranyl-geranyl diphosphate (geranylgeranylation). In CAAX, C indicates a cysteine residue, A indicates an aliphatic amino acid and X indicates variable amino acids [29] (Figure S3). Considering these data, we hypothesize that Tri14 could have some role in the uptake of a lipid-like metabolite related to trichothecene biosynthesis. However, additional studies would be needed to test this hypothesis.

#### Effect of *tri14* Deletion on Resistance to Farnesol

Based on the Tri14 structure analysis, the effect of *tri14* deletion on resistance to farnesol was carried out. The results of this analysis indicated that *tri14* deletion and overexpression did not affect resistance to farnesol at concentrations up to 1 mM, the highest concentration analyzed (Figure S4).

### 2.8. tri14 and HA Resistance

**Figure 6:**
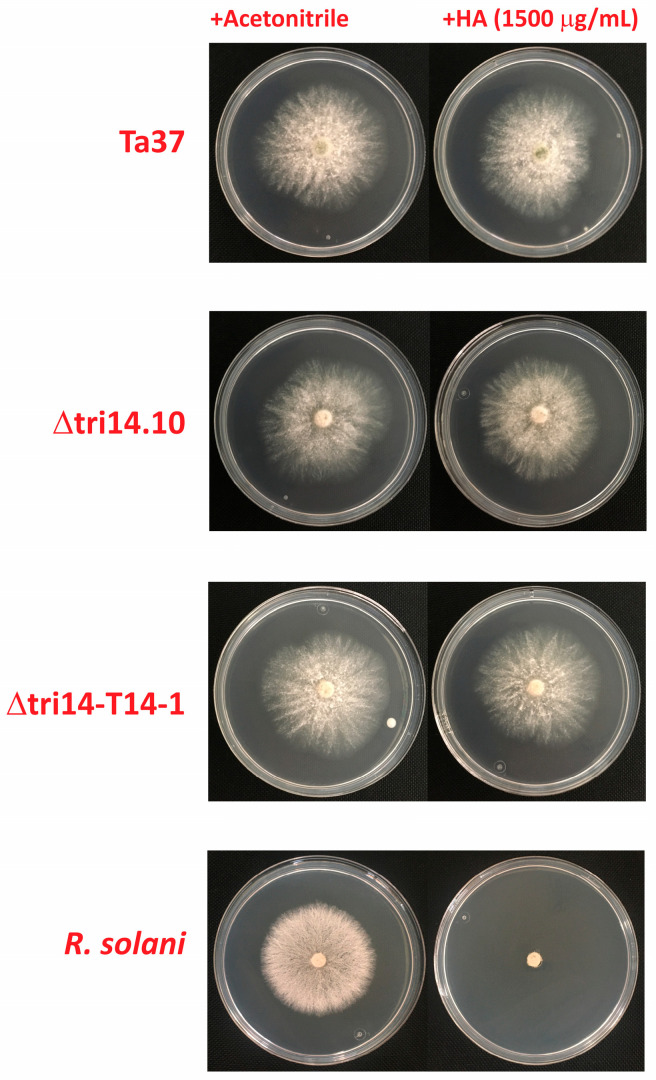
HA self-protection assay. Resistance against purified HA by wild-type Ta37 strain and two of the transformants analyzed previously. Note that no differences in growth were observed in plates amended with HA (**right plates**) in comparison with control plates amended with acetonitrile, the solvent used to dissolve HA (**left plates**). Also note at the bottom of the figure the inhibitory effect of HA on *R. solani* growth, which confirmed that the batch of HA was active.

Initial analyses showing that *tri14* is conserved in all trichothecene-producing fungi led to the hypothesis that the gene confers resistance/self-protection against the toxic effects of trichothecenes. Although the recent finding that Tri14 catalyzes the cyclization of isotrichodiol and isotrichotriol casts doubt on this hypothesis, we deemed it still worthwhile to test it. Therefore, we grew wild-type (Ta37), *tri14* mutant (Δtri14.10) and *tri14* add-back transformants of *T. arundinaceum* in the presence and absence of 1500 μg/mL HA. The results of this analysis revealed that HA did not cause growth inhibition in any of the strains examined (Figure 6). These results indicate that Tri14 does not impact the resistance of *T. arundinaceum* to HA under the conditions examined.

### 2.9. tri14 and Expression of ROS Detoxification Genes

**Figure 7:**
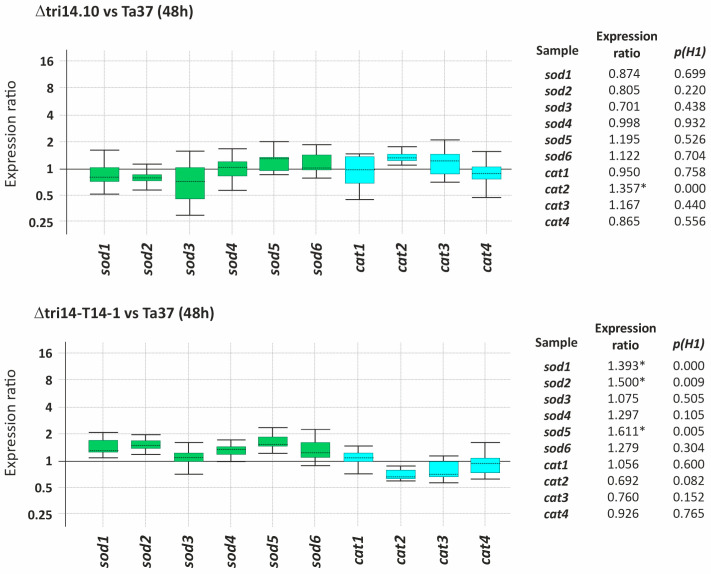
Effect of *tri14* gene deletion on expression of *T. arundinaceum* genes related to ROS detoxification. qPCR Ct values and expression ratios were analyzed as described in the legend of Figure 4. Statistically significant values (p(H1) < 0.05) are indicated with an asterisk at the right panels.

A previous study provided evidence that Tri14 plays a role in ROS tolerance in *F. graminearum* [15]. To investigate whether the same is true in *T. arundinaceum*, we used quantitative PCR to compare expression of two types of genes associated with ROS detoxification (*cat* = catalase-encoding genes; *sod* = superoxide dismutase-encoding genes) in wild-type (Ta37), *tri14* mutant (Δtri14.10) and *tri14* add-back strains of the fungus following exposure to 0, 5 and 25 mM hydrogen peroxide (H_2_O_2_). The results of the analysis indicate that *tri14* deletion did not significantly affect the expression of *cat* or *sod* genes, except that *cat2* was slightly upregulated in the *tri14* mutant compared to the wild type. In the *tri14* add-back strains, there was a moderate level of upregulation of the six *sod* genes analyzed, but the upregulation was not statistically significant for three of the genes (Figure 7).

These data indicate that Tri14 does not have a marked effect on the expression of the *cat* and *sod* genes associated with ROS detoxification. It is important to note, however, that *tri14* deletion resulted in a significant reduction in HA production in *T. arundinaceum*. To determine whether the limited effect of Tri14 on *cat* and *sod* gene expression was due to the change in HA production, we assessed the expression of the *cat* and *sod* genes in a *tri5* deletion mutant (strain Δtri5.3) of *T. arundinaceum* that was generated in a previous study [4]. Because *tri5* encodes the terpene synthase (Tri5) that catalyzes the first committed step in trichothecene biosynthesis, the *tri5* deletion mutant does not produce any trichothecenes or trichothecene biosynthetic intermediates. Most *cat* and *sod* genes analyzed were upregulated in the *tri5* mutant compared to the wild-type progenitor strain (Ta37). However, the *tri5* add-back transformant, the strain Δtri5-T5-24, exhibited a similar effect (Figure S5). Thus, the effect observed in the *tri5* deletion mutant was almost certainly not caused by a loss of HA production. Thus, the altered expression of *cat* and *sod* genes in the *tri14* mutant was unlikely to be caused by a reduction in HA production in the mutant.

### 2.10. tri14 and Resistance to H_2_O_2_

We also tested the effect of *tri14* on resistance to ROS by growing *T. arundinaceum* strains on a solid medium amended with H_2_O_2_ at 5 mM and 25 mM to induce oxidative stress in the cultures of the wild-type, *tri14* mutant and *tri14* add-back strains, as well as a Ta37-derived *tri14* overexpression strain. The results of the analysis indicated that neither the deletion nor the overexpression of *tri14* affected ROS resistance under the conditions employed in this study (Figure S6).

### 2.11. tri14 Deletion and Metabolite Profile

**Figure 8:**
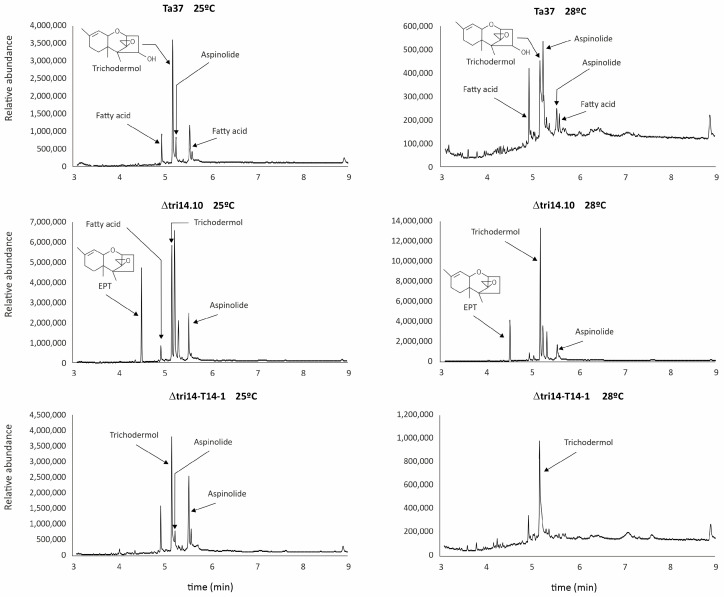
Gas chromatography–mass spectrometry (GC-MS) chromatograms of liquid culture samples of the wild-type (Ta37), *tri14* deletion mutant (∆tri14.10) and *tri14* add-back (∆tri14-T14-1) strains grown at 25 °C (**left**) or 28 °C (**right**) for 7 days in YEPD medium.

Metabolite production profiles in the wild-type (Ta37), *tri14* mutant (Δtri14.10) and *tri14* add-back transformant (Δtri14-T14-1) strains were assessed by gas chromatography–mass spectrometry (GC-MS) analysis (Figure 8). The strains were grown in liquid YEPD medium at 25 °C and 28 °C for 7 days. Based on previous reports, GC-MS analysis is a suitable method to detect a wide variety of secondary metabolites, e.g., trichothecene and aspinolide analogs. At 25 °C, the wild type produced high levels of trichodermol and lower levels of aspinolides and fatty acids. The *tri14* mutant produced a different aspinolide and significant levels of EPT (12,13-epoxytrichothec-9-ene) (Figure 8). By contrast, the *tri14* add-back strain had a metabolic profile that was more similar to the wild-type profile, with a balanced production of trichodermol and two aspinolides. At 28 °C, the metabolite profiles shifted in all strains. In the wild type, trichodermol and aspinolide production was lower at 28 °C compared to that at 25 °C, but the two aspinolides were detectable and the aspinolide/trichodermol ratio was increased. The *tri14* mutant displayed an enhanced production of trichodermol at 28 °C compared to at 25 °C, while production of EPT and aspinolide analogs remained similar at both temperatures. Conversely, in the *tri14* add-back transformant, only moderate levels of trichodermol were produced at both temperatures (Figure 8).

## 3. Discussion

This study investigated the role of the gene *tri14* in the biology of *Trichoderma arundinaceum*, specifically the effect of *tri14* on trichothecene production, antifungal activity, *tri* gene expression, and oxidative stress response. Previous studies that showed conservation of *tri14* in all trichothecene-producing fungi have suggested that the gene has an important function in trichothecene biosynthesis. However, the previous studies also revealed that *tri14* deletion did not affect trichothecene toxin production in culture. That is, production of deoxynivalenol was not affected in *tri14* deletion mutants of *F. graminearum* [15,22], and production of trichodermin was not affected in a *tri14* deletion mutant of *T. brevicompactum* [23]. In marked contrast, the *tri14* deletion mutant of *T. arundinaceum* generated in the current study exhibited a substantial reduction (69%) in the production of HA with a concomitant increase in EPT level when the mutant was grown in culture. The reduction in HA and increase in EPT levels were caused by *tri14* deletion, which was confirmed by increased HA production and a reduction in EPT levels in *tri14* add-back strains. Why the effect of *tri14* deletion on trichothecene production in *T. arundinaceum* differed from the effect in *F. graminearum* and the closely related species *T. brevicompactum* is unclear and warrants further investigation.

This reduction in HA production in the *T. arundinaceum tri14* deletion mutant correlates with a reduction in the antifungal capacity, as observed in the antifungal assays against *R. solani*. This antifungal capacity was restored in the add-back transformants of Δtri14.10 (Figure 5, Table S3).

The *tri* gene expression study revealed that in the absence of *tri14*, only the expression of *tri23* was increased, while expression of most other *tri* genes was not significantly affected. In previous work with *F. graminearum*, the expression of other *tri* genes was not significantly affected in a *tri14* deletion strain [15], which is consistent with our results. Nevertheless, in the *tri14* add-back mutants, most *tri* genes are significantly upregulated compared to wild-type strain, especially *tri4* and *tri3*, including the transcription factors encoding genes, *tri10* and *tri6* [4,18].

A noteworthy result related to the effect of Tri14 on trichothecene biosynthesis is the change in the secondary metabolites’ profiles. Cultures of the *tri14* deletion mutant accumulated the trichothecene parent compound EPT, but cultures of the wild-type and the *tri14* add-back mutant did not. During trichothecene biosynthesis, EPT is transformed into trichodermol by the monooxygenase encoded by *tri22*, and later, an acetyl transferase encoded by *tri3* completes the conversion to HA [2,4,18]. Trichodermol is the major metabolite observed in the wild-type (Ta37) and the *tri14* add-back (Δtri14-T14-1) metabolic profiles. This indicates that HA precursors are accumulating in the absence of *tri14*, which reinforces the role of this gene in trichothecene biosynthesis.

The in silico analysis of Tri14 revealed the abundance of β-lamina and a 3D structure of β-barrel, similar to what has been found for Tri14 proteins in *F. sporotrichioides*, *F. graminearum*, *T. arundinaceum* and *T. brevicompactum* (Figure S3). This conformation is typical for fatty acid binding proteins. Moreover, two CAAX motifs were found in the amino acid sequence, which indicate a possible function related to prenylation. Therefore, Tri14 could be involved in the processes of transportation of lipophilic precursors essential to trichothecene biosynthesis, such as farnesyl diphosphate (FDP). Similarly, structural modifications, particularly by triphenylphosphonium cation (TPP+), have been shown to enable organelle-specific or cellular targeting in mammal cells [30,31]. Assays to determine Tri14’s role in resistance to farnesol indicated that *tri14* deletion or overexpression do not affect resistance to farnesol (Figure S4).

In the previous study of *T. brevicompactum*, *tri14* deletion affected the expression of some trichothecene biosynthetic genes but not others. That is, the RT-PCR analysis revealed a dramatic increase in the expression of *tri22* but no effect on *tri4* and *tri5* expression. However, the production yield of trichodermin was not significantly affected [23].

Although previous studies in *F. graminearum* suggest a role of Tri14 in protecting the organism from the plants’ defensive oxidative stress induction [15], our experiments did not show significant differences in resistance to H_2_O_2_ between the wild-type strain and the *tri14* mutants in growth assays, and we only found slight differences when the ROS defense genes’ expression was analyzed. In this case, 6 *sod* and 4 *cat* genes’ expression was assayed by RT-qPCR, and only the *cat2* gene was slightly upregulated in the *tri14* deleted mutant, and in the Δtri14-T14-1 add-back mutant, three *sod* genes were slightly upregulated as well. These results do not indicate a clear role of *tri14* in oxidative stress response.

Future studies should explore whether Tri14 directly interacts with enzymes in the biosynthetic pathway or if it modulates the transcriptional regulation of *tri* genes through interactions with transcription factors. Additionally, X-ray crystallography studies could provide detailed information on the structure of Tri14 and its potential binding to lipids. Finally, co-immunoprecipitation or yeast two-hybrid experiments could help determine if Tri14 interacts with regulatory proteins such as Tri6 or Tri10.

In the current study, we sought to determine the impact of Tri14 on multiple facets of *T. arundinaceum* biology, including trichothecene biosynthesis, antifungal activity, resistance to HA and oxidative stress. The results provide a foundation for the further characterization of the effect of Tri14 on *T. arundinaceum* and other trichothecene-producing fungi. The finding that *tri14* deletion reduces trichothecene production in *T. arundinaceum* but not in *T. brevicompactum* or *F. graminearum* provides evidence that the impact of *tri14* differs among trichothecene-producing fungi. The cause of such differences remains to be determined.

## 4. Materials and Methods

### 4.1. Strains Used and Culture Conditions

*Trichoderma arundinaceum* IBT 40837 (=Ta37) was used as the target strain to delete *tri14* and as the wild-type control strain in the current study. The plant pathogenic fungus *Rhizoctonia solani*, strain R43, stored at the “Plant and Pest Diagnostic Laboratory Collection” of the University of León (Spain), was used in the antifungal assays and also as a control in the HA self-protection assay.

All fungal strains were maintained on potato dextrose agar medium (PDA), prepared from PDB broth (Becton Dickinson Co., Franklin Lakes, NJ, USA) amended with 2.5% agar (Oxoid Ltd., Basingstoke, UK). Sporulation of *Trichoderma* strains occurred after 5–7 days of incubation at 28 °C in the dark. *R. solani* was also grown on PDA plates in the same conditions as those indicated above.

Cultures for HA production were carried out as previously reported [4,18].

### 4.2. Plasmid Construction

#### 4.2.1. Construction of Plasmid pΔtri14 for *T. arundinaceum tri14* Deletion

PCR reactions were carried out with oligonucleotide pairs TRI14_5r_R_BamHI/TRI14_5r_F_SmaI and TRI14_3r_R_SmaI/TRI14_3r_F_SalI (Table S1), and *T. arundinaceum* genomic DNA was used as template to amplify 1039 bp and 1068 bp fragments corresponding to the 5′ and 3′ *tri14*-flanking regions, respectively. These amplified bands were phosphorylated with T4-polynucleotide kinase (Thermo Fisher Scientific, Foster City, CA, USA) extracted from agarose gels and subcloned in plasmid pBluescript KS+ (Stratagene, San Diego, CA, USA), which was previously linearized with *Eco*RV and dephosphorylated with alkaline phosphatase (Thermo Fisher Scientific), following routine procedures to obtain plasmids pB_T14_5R (4012 bp) and pB_T14_3R_b (4041 bp). Plasmid pB_T14_5R was digested with *Sma*I-*Bam*HI, and the fragment corresponding to the 5′ *tri14*-flanking region (1064 bp) was gel-purified and cloned into pB_T14_3R_b, which had been previously linearized with the same enzymes. The resulting plasmid, pB_T14_3R_5R (5059 bp), was finally linearized with *Sma*I, dephosphorylated and ligated to the hygromycin resistance cassette (2710 bp), which was isolated from pAN7-1 [32] by digestion with *Ecl*136II-*Hin*dIII and treated with Klenow fragment (Thermo Fisher Scientific) to obtain plasmid pΔtri14 (7774 bp) (Figure 1). This plasmid was linearized with *Apa*I prior its transformation in *T. arundinaceum* protoplasts.

#### 4.2.2. Construction of *T. arundinaceum tri14* Overexpression Plasmid

The *tri14* ORF was amplified from *T. arundinaceum* genomic DNA using oligonucleotides TARUN_T14_ATG and TARUN_T14_end (Table S1) and Q5 high-fidelity DNA polymerase (New England Biolabs, Ipswich, MA, USA). The amplified fragment (1151 bp) was phosphorylated with T4-polynucleotide kinase and ligated to pTAcbh plasmid [18] previously linearized with *Nco*I, filled with Klenow fragment and dephosphorylated with alkaline phosphatase. The resulting plasmid pTC_TARUN_T14a (6490 bp) was linearized with *Hin*dIII, filled with Klenow fragment, dephosphorylated and ligated to the 1591 bp fragment that includes the bleomycin/phleomycin resistance cassette (*ble*), which was isolated from pJL43b1 [18] by digestion with *Hin*dIII, filled with Klenow and again digested with *Ecl*136II. The final plasmid pTC_TARUN_T14a_ble_a (8089 bp) (Figure 1) was linearized with *Nar*I prior to protoplast transformation.

### 4.3. Transformation Procedures

Ta37 and its derived strains were transformed following a protoplast-based procedure as previously reported [18], using *Trichoderma* regeneration medium (0.1% yeast extract (Sigma Aldrich, St. Louis, MO, USA), 0.1% NZ-Amine (Sigma Aldrich), 27.4% sucrose (Sigma Aldrich) and 1.6% agar (Becton Dickinson Co., Franklin Lakes, NJ, USA)) amended with 250 mg/mL of hygromycin for selection of *tri14*-deleted transformants, or 1M sorbitol-Czapek medium amended with 100 mg/mL phleomycin for selection of *tri14* overexpression transformants.

### 4.4. Metabolomics Characterization

#### 4.4.1. HA Purification and Quantification

HA was quantified by HPLC from 48 h PBD liquid cultures as previously described [18]. Thus, 3.5 mL were extracted two-fold with ethyl acetate, then solvent was evaporated in a SpeedVac^TM^ concentrator (Thermo Fisher Scientific) and resuspended into 350 μL of acetonitrile [18]. For self-protection studies, HA was purified as indicated above from 48 h PDB grown cultures of wild-type Ta37 strain and finally diluted to 1500 μg/mL in acetonitrile [18].

#### 4.4.2. Gas Chromatography–Mass Spectrometry (GC-MS) Analysis

The strains were grown in liquid YEPD medium at two different temperatures, 25 °C and 28 °C, for 7 days. Cultures were extracted with 3 mL of ethyl acetate and analyzed by GC-MS as previously described [3,18].

### 4.5. Antifungal Assays

Antifungal assays were implemented to analyze the different strains and metabolites analyzed in the current study against *R. solani*. These antifungal assays were performed on cellophane membranes as previously described [4,18].

#### 4.5.1. HA Self-Inhibition Activity

A volume of 60 μL of a 1500 μg/mL HA solution was dispensed into a 7 mm hole in the center of a 90 mm diameter Petri dish containing 20 mL of PDA (1% agar) medium. The solution was allowed to diffuse for 24 h at 4 °C. After this time, a plug of a 1-week-old PDA culture of the strain to be analyzed was placed in the 7 mm hole and incubated at 28 °C for 1 week. The effect of HA on growth was determined by measuring the diameter of the colony, in comparison with control plates in which the solvent (acetonitrile) without HA was applied.

#### 4.5.2. Farnesol Resistance

Concentrations up to 1000 μM of farnesol were used to analyze the effect of *tri14* deletion on the resistance of transformants to this compound. A 90 mm diameter Petri dish containing 20 mL of PDA (1% agar) medium with or without farnesol was used to place a 7 mm plug of a 1-week-old PDA culture of the strain to be analyzed and incubated at 28 °C for 1 week. The effect of farnesol on growth was determined by measuring the diameter of the colony, in comparison with control plates without farnesol.

### 4.6. Quantitative PCR (qPCR)

Extraction and purification of RNAs from fungal mycelia, as well as the qPCR reactions, were performed as previously described [15,18]. Oligonucleotides used for qPCR analysis of Ta37 actin, *tri3*, *tri4*, *tri5*, *tri6*, *tri10*, *tri12*, *tri18* and *tri17* in previous studies are described in Table S1. Furthermore, oligonucleotides used for analysis of *T. arundinaceum tri14*, *tri23*, and superoxide dismutase- and catalase-encoding genes were designed for the current study (Table S1). qPCR reactions were carried out in a STEP ONE (Applied biosystems, Foster City, CA, USA) device following manufacturer’s instructions, and results were analyzed using REST^©^ 2009 (version 2.0.11) software [27,28].

### 4.7. Genome Sequencing and Analysis

Genome sequence of the mutant strain for the current study was generated by the company Macrogen (Seoul, Korea; https://dna.macrogen.com, accessed on 10 November 2021) using an Illumina platform, and sequence assembly was carried out by the SPAdes (v3.15.0) assembler [33]. The unassembled reads obtained as result of the whole-genome shotgun of *tri14*-deleted mutant were deposited at the Sequence Read Archive (SRA) of the DDBJ/ENA/GenBank under the accession number SRRR32457847.

## Figures and Tables

**Table 1:** HA production quantified by HPLC from 48 h PDB culture filtrates of the wild-type (Ta37), *tri14* deletion mutant (Δtri14.10) and *tri14* add-back strains (Δtri14-T14-1 and Δtri14-T14-3) of *T. arundinaceum*.

Strain	HA Production μg/mL	% HA vs. Ta37 *
Ta37	229.67 ± 7.92	100
Δtri14.10	71.16 ± 3.16	31
Δtri14-T14-1	147.58 ± 4.12	64
Δtri14-T14-3	270.94 ± 33.98	118

*n* = 2. * Values are the % of HA levels produced by each strain relative to Ta37 (wild-type progenitor strain), with the levels produced by Ta37 for each experiment being taken as 100%.

**Table 2:** HA production quantified by HPLC from 48 h PDB culture filtrates from the wild-type (Ta37) and three Ta37-*tri14* overexpression strains (Ta37-T14-2, Ta37-T14-3, and Ta37-T14-5).

Strain	HA Production μg/mL	% HA vs. Ta37 *
Ta37	239.75 ± 14.24	100
Ta37-T14-2	218.51 ± 16.76	91
Ta37-T14-3	291.25 ± 7.11	121
Ta37-T14-5	278.67 ± 1.63	116

*n* = 2. * Values are the % of HA levels produced by each strain relative to Ta37 (wild-type progenitor strain), with the levels produced by Ta37 for each experiment being taken as 100%.

## Data Availability

Data presented in this study are available in this manuscript. In addition, unassembled reads obtained as result of the whole-genome shotgun of *tri14*-deleted mutant were deposited at the Sequence Read Archive (SRA) of the DDBJ/ENA/GenBank under the accession number SRRR32457847.

## Supplementary Materials

The following supporting information can be downloaded at: https://www.mdpi.com/article/10.3390/toxins17090427/s1. Table S1: Oligonucleotides used in the present work. Table S2: HA production quantified by HPLC from 48 h PDB culture broths. Table S3: Percentages of radial growth inhibition (RI) of *R. solani* by strains analyzed in the present work. Figure S1: PCR analysis of selected *T. arundinaceum* transformants obtained with plasmid pΔtri14. Figure S2: Agarose gel electrophoresis of PCR reactions carried out to detect transformants that have incorporated the different constructs designed for overexpression of *tri14* gene. Figure S3: Predictive analysis of *T. arundinaceum* Tri14 tertiary structure. Figure S4: Effect of *tri14* gene deletion and overexpression on resistance to farnesol. Figure S5. Effect of *tri5*-deletion on expression of *T. arundinaceum* genes related to ROS detoxification. Figure S6: Growth under oxidative stress conditions.

## Abbreviations

The following abbreviations are used in this manuscript:

Ta37	*Trichoderma arundinaceum* IBT 40837
EPT	12,13-epoxytrichothec-9-ene
DON	Deoxynivalenol
ROS	Reactive oxygen species
HA	Harzianum A
HPLC	High-performance liquid chromatography
TLC	Thin-layer chromatography
GC-MS	Gas chromatography–mass spectrometry
RT-PCR	Reverse transcription polymerase chain reaction
