# Supplementary material for: Role of the Gene tri14 in Biosynthesis of the Trichothecene Toxin Harzianum A in Trichoderma arundinaceum
Source: Toxins (Basel). 2025 Aug 26;17(9):427. doi: 10.3390/toxins17090427 (PMC12474229; doi:10.3390/toxins17090427)
Supplement: Supplementary file 1 [file toxins-17-00427-s001.zip › toxins-3770029-supplementary.pdf]

Supplementary Material: Role of the Gene *tri14* in Biosynthesis of the Trichothecene Toxin Harzianum A in *Trichoderma arundinaceum*

Natalia Martínez-Reyes, Rosa E. Cardoza, Susan P. McCormick, Guixia Hao, Joaquín Rodríguez-Fernández, Robert H. Proctor and Santiago Gutiérrez

Table S1. Oligonucleotides used in the present work.

**Oligonucleotide Name**	**Sequence 5´-3´**
**Construction of p *tri14* to delete *Trichoderma arundinaceum tri14* gene.**	
TRI14_5r_R_BamHI (1039 bp)	ggatccCATATACGGAGTACCAGG
TRI14_5r_F_SmaI	cccgggCGAGTGATTCAAGAACGC
TRI14_3r_R_SmaI (1068 bp)	cccgggAGTGCTTGACTCAGGTCA
TRI14_3r_F_SalI	gtcgacGCGCTAATGGATGTAGC
**Oligonucleotides to amplify an internal fragment to *tri14* gene.**	
T14_IF_Ct (820 bp)	TCGTGATATGGCGTCTGA
T14_IF_Nt	CAGGTGTTACTGAGCTCA
Ptadir (993 bp, when used with T14_IF_Ct)	CACTGCAGTCCACATTGA
**Oligonucleotides to analyze transformants for detection of *tri14* gene-deleted mutants.**	
tri14-5r_rr	GCAAGCATCAATTCAGCC
tri14-3r_ff	CCTTGTAAGGATACGGAG
**Amplification of *T. arundinaceum tri14***	
TARUN_T14_ATG (1151 bp)	CAACCACAGGTGTTACTG
TARUN_T14_end	TCATGCTACAAGAGCATC
**Oligonucleotides used for qPCR analyses**	

**Gene**	**Oligo name**	**Sequence 5´-3´**
actin	aactinF	ACTGGGACGACATGGAGAAG
	aactinR	GGCCTGGATGGAGACATAGA
tri3	3woiF	TTACAGTGGGGTACGGAAGC
	3woiR	CGAGGTAATCCCAATCCAGA
tri4	4woiF	ACTGTCGGAGCTTTCGATGT
	4woiR	GCTGAGAAGGCTCCATCAAG
tri5	5woiF	TGTGTCGGCTCTTCAATCTG
	5woiR	GCTTATCGAGAGCCTGATCG
tri6	6woiF	GGTGCAAATCTACCGCTCTC
	6woiR	CGGGTTGTGTTTAGCCTCAT
tri10	10woiF	GCCTAATCCAGATGGACCAA
	10woiR	CAATGCAGAGGTTGCGTAGA
tri12	12woiF	CTATGGCCTTGGGGGTTATT
	12woiR	ACAGCGGCTGAGCTAACATT
tri18	T18qF	CCTGTTGACGGGCGTTTATA
	T18qR	TTTCGTTGATGGCCCAAGAC
tri17	tri17F	GACTCCCTTGCACAGTTTGA
	tri17R	ACAGCAGCGTCTTCAATTCC

tri14	14woiF	ACGCCAAATCCTTTGCATAC
	14woiR	GCTCAGCATCAGCATAACCA
tri23	P450qF	TGGCTCTGCCGATTGGAAAA
	P450qR	CATTGTGATGCTTGCCGAGT
sod1	TaSOD1F	ACTTCAACCTTGGCGCATTT
	TaSOD1R	TCCGAAGTGATCTTGCCGTA
sod2	TaSOD2F	GACGTCCCTTCACAGATTGC
	TaSOD2R	GATGGTGACAGGTTCTCCCA
sod3	TaSOD3F	CTCCATGGCTCACAACAACC
	TaSOD3R	GAGGAGAAGGAGGCTTCGAG
sod4	TaSOD4F	ATACGTCAACGGCTTCAACG
	TaSOD4R	GTTCTCCCAGAAGAGCGAGT
sod5	TaSOD5F	TCCACATCCACACCTTTGGT
	TaSOD5R	AGAGCCGTGAGTCTTGTTGA
sod6	TaSOD6F	GTCCGGAGGAGAGAAGGAAG
	TaSOD6R	TGGCCAATGACTTCCCAGAT
cat1	TaCAT1F	CCCAGGTTCACCTCAAGACT
	TaCAT1R	CAGCGGAACTTCTCAGCTTC
cat2	TaCAT2F	TGAGCCGACTGGGTGTTAAT
	TaCAT2R	GGCTGTGCTTCGAATCTGTT
cat3	TaCAT3F	TCGAGAAGGGAGACTACCCA
	TaCAT3R	TTTCTCATACGGCCAGGTGT
cat4	TaCAT4F	GACGTTCTCCAGATGCCAAC
	TaCAT4R	TGTGTCTCCCACAACTCCTC

Table S2. HA production quantified by HPLC from 48 h PDB culture broths of Ta37 wild-type strain, Dtri14.10 mutant, and transformants of Dtri14.10 overexpressing *T. arundinaceum tri14* (Dtri14-T14-#)

Strain	HA production mg/mL
**Ta37**	229.67
**tri14.10**	71.16
**tri14-T14-1**	147.58
**tri14-T14-2**	171.29
**tri14-T14-3**	270.94
**tri14-T14-5**	207.23
**tri14-T14-6**	181.59

Table S3. Percentages of radial growth inhibition (RI) of *R. solani* by strains analyzed in the present work after 15 days of incubation after pathogen´s plug disposal

		**% RI/% RI in Ta37***
**Ta37**	$51.64^{a} \pm 3.84$	100
**tri14.10**	$29.09^{b} \pm 4.23$	56.33
**tri14-T14-1**	$43.23^{a} \pm 10.08$	83.71
**tri14-T14-2**	$48.05^{a} \pm 4.87$	93.04
**tri14-T14-3**	$53.27^{a} \pm 1.05$	103.15
**tri14-T14-5**	$46.83^{a} \pm 5.47$	90.68
**tri14-T14-6**	$53.29^{a} \pm 1.69$	103.19

n= 3, ANOVA.

[a,b] On each column, values followed by different superscript letters are significantly different ($p < 0.05$).

*This column includes the ratio of radial growth inhibition (RI) for each strain versus that for the wild-type strain, assigning a ratio of 100% to the values observed for the wild-type strain.

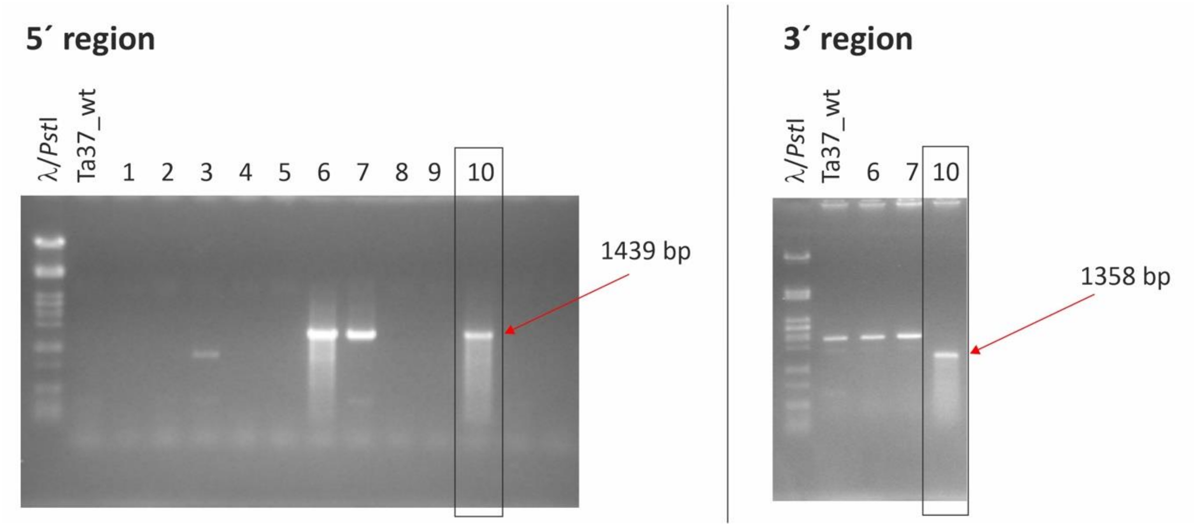

Figure S1. PCR analysis of selected *T. arundinaceum* transfomants obtained with plasmid pΔtri14 using primer pairs tri14-5r_rr/TtrpC-d (left panel), and PgpdA-d/tri14-3r_ff (right panel) that amplify 1439 bp and 1358 bp DNA fragments corresponding to the 5´ and 3´ extremes of the recombination cassette designed for *tri14* deletion. *λ*/*PstI* = DNA from phage lambda digested with *PstI*, used as molecular size marker. Transformant #10 exhibited the expected PCR pattern, which indicates replacement of *tri14* with *hph*.

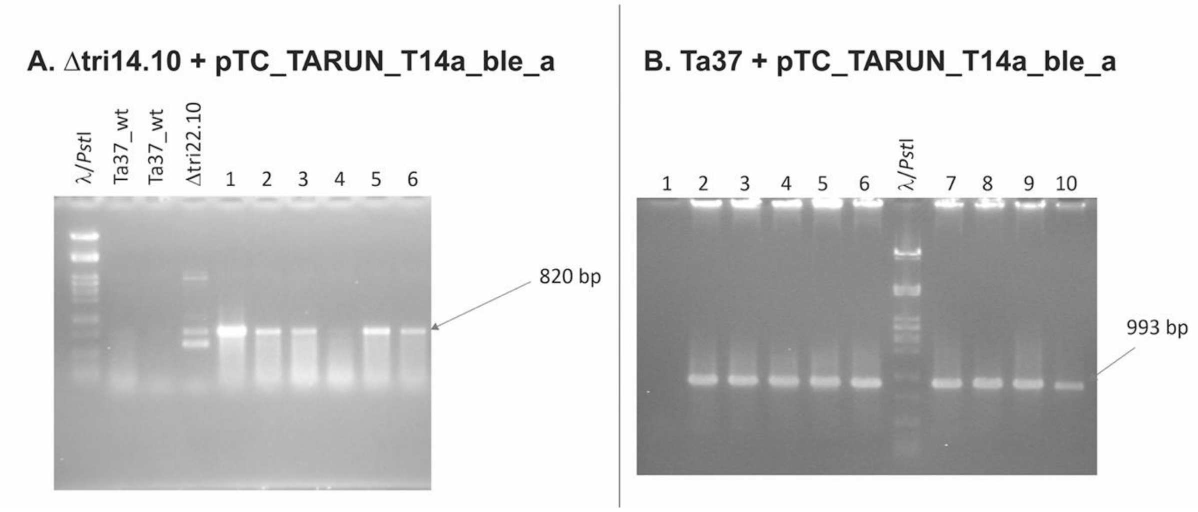

Figure S2. Agarose gel electrophoresis of PCR reactions carried out to detect transformants that have incorporated the different constructs designed for overexpression of: **A**. *T. arundinaceum tri14* in Dtri14.10 mutant, or **B**. *T. arundinaceum tri14* gene in Ta37.
Size of the expected fragments for each PCR reaction are shown at the right of each agarose gel. Oligonucleotide pairs used in the different analyses are indicated in the text. l/*Pst*I, lambda phage DNA digested with the endonuclease *Pst*I, used as a DNA size marker.
Note that the intensity of the 820 bp band in transformant 4 in panel A was very weak. Thus, this transformant was discarded for further studies.

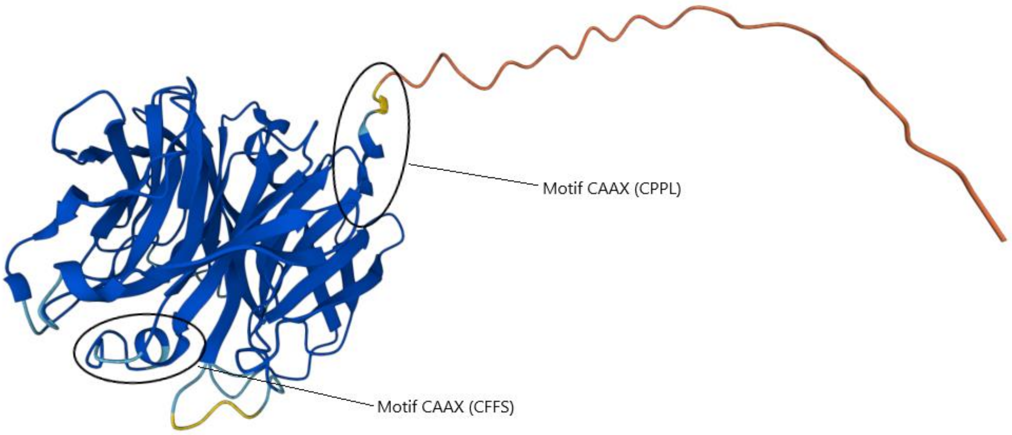

Figure S3. Predictive analysis of *T. arundinaceum* Tri14 tertiary structure, using AlphaFold software (https://alphafold.ebi.ac.uk). CAAX domains are also pointed.

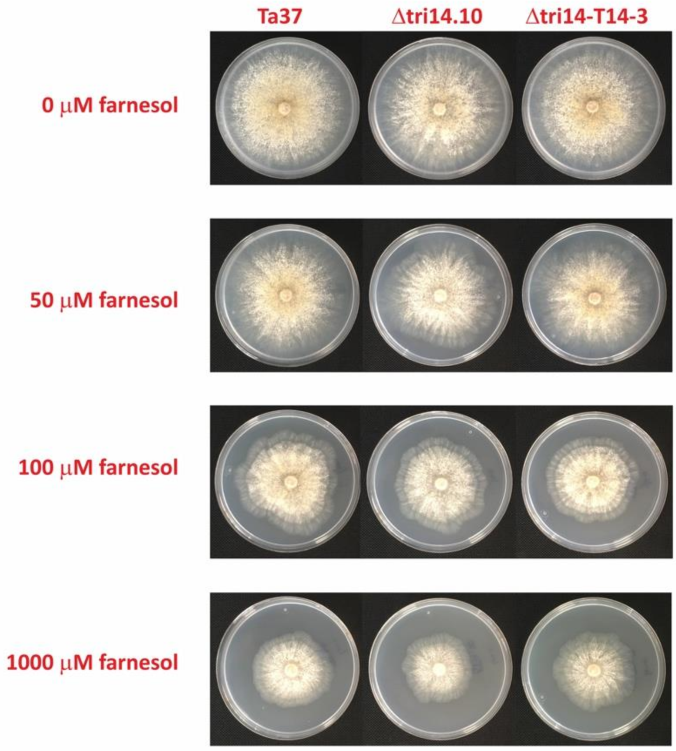

Figure S4. Effect of *tri14*-gene deletion and overexpression on resistance to farnesol

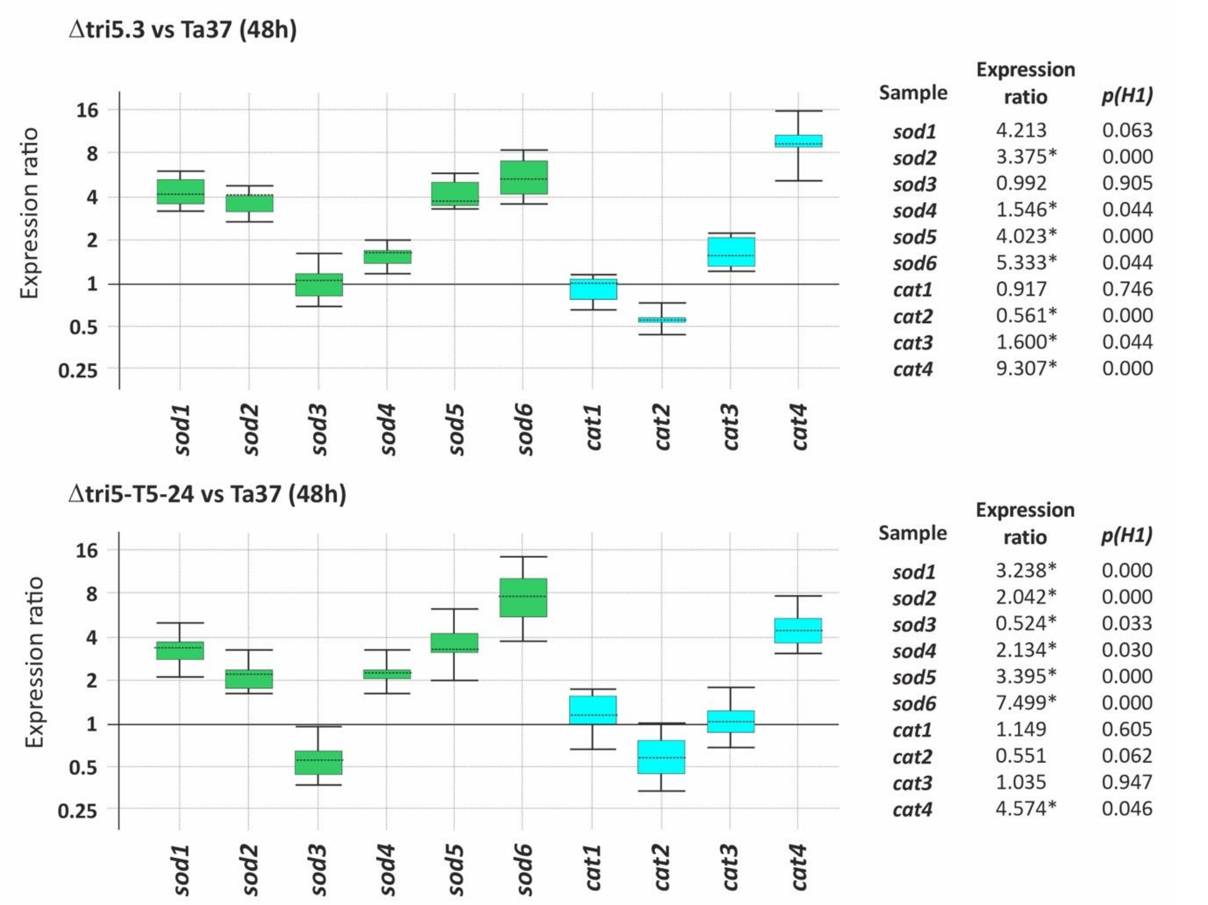

Figure S5. Effect of *tri5*-deletion on expression of *T. arundinaceum* genes related to ROS detoxification. qPCR Ct values and expression ratios were analyzed as described in the legend of Figure 4. Statistically significant values (p(H1) < 0.05) are indicated with an asterisk at the right panels.

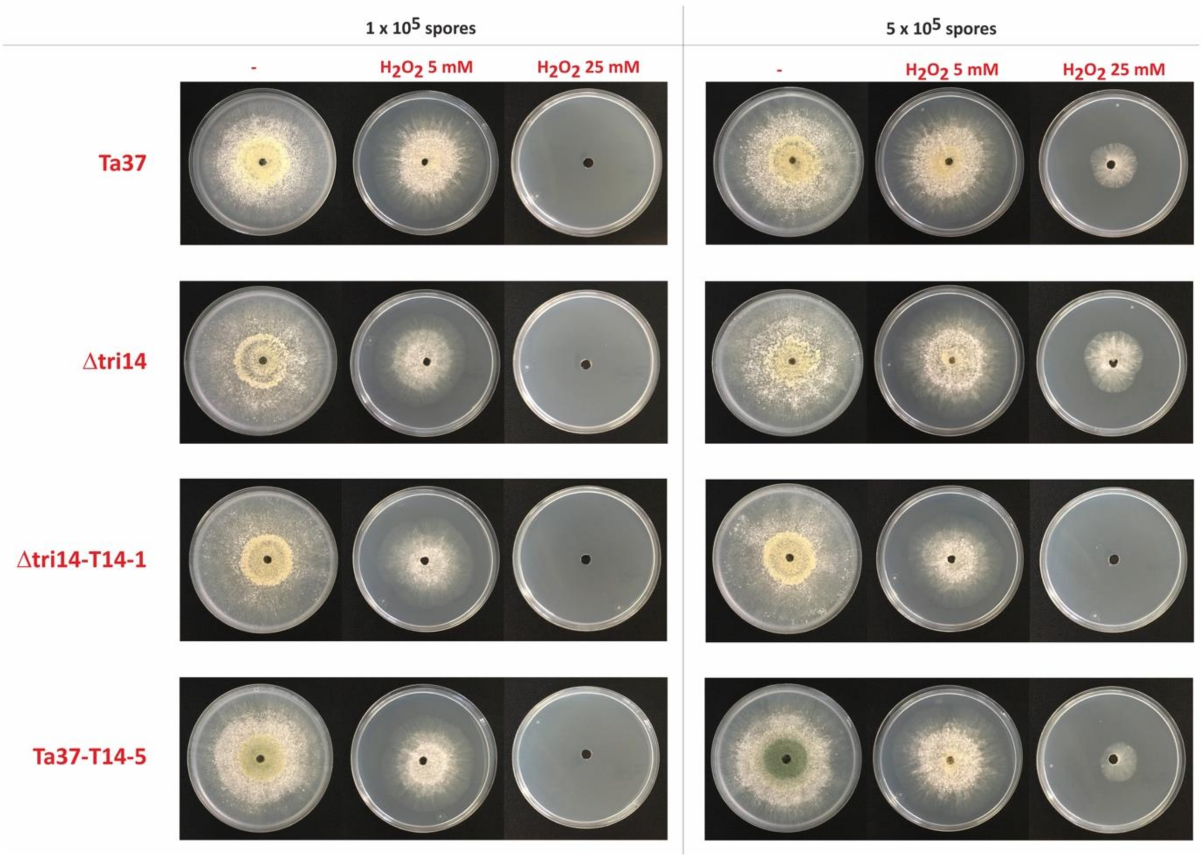

Figure S6. Growth under oxidative stress conditions. The wild type (Ta37), *tri14* deletion mutant (Δtri14.10), *tri14* add-back, and Ta37-*tri14* overexpression (Ta37-T14-5) strains were grown for 5 days on solid media treated with 0, 5 or 25 mM H_2O_2. Plates were inoculated with 1 x 10^5 (left) and 5 x 10^5 (right) conidia.
